# Supplemental Nutrition Assistance Program healthy eating incentives and fruit and vegetable spending across the month

**DOI:** 10.1017/S1368980024001770

**Published:** 2024-09-26

**Authors:** Joel Cuffey, Candace Lantz, Kara Newby, Alicia Powers

**Affiliations:** 1 Department of Agricultural Economics and Rural Sociology, Auburn University, Auburn, AL, USA; 2 Graduate student, Food and Resource Economics Department, University of Florida, Gainesville, FL, USA; 3 Hunger Solutions Institute, Auburn University, Auburn, AL, USA

**Keywords:** Supplemental Nutrition, Assistance Program incentives, SNAP cycle, Produce expenditure, Food assistance policy

## Abstract

**Objective:**

To estimate how incentives that encourage healthy eating among Supplemental Nutrition Assistance Program (SNAP) participants impact intra-monthly variation in fruit and vegetable spending.

**Design:**

We used transaction data from three Alabama grocery stores participating in a programme that offered dollar-matching coupons for fresh produce. For each store, we calculated daily spending on fresh produce out of SNAP benefits and daily incentive coupon redemptions. We compared total daily spending on fresh produce and daily coupon redemptions on days over which SNAP benefits are distributed in Alabama with spending and redemption on days at the end of the month with no SNAP distribution.

**Setting:**

SNAP and incentive transactions in three Alabama grocery stores.

**Participants:**

SNAP participants purchasing fruit and vegetables April 2023–July 2023.

**Results:**

Daily spending with SNAP on produce dropped by 38% at the end of the month. Incentive coupon redemption did not significantly drop at the end of the month. The share of total SNAP spending going to fresh fruits and vegetables increased by two percentage points and the share of fresh fruits and vegetables spending coming from redemptions increased by ten percentage points at the end of the month.

**Conclusions:**

SNAP households may use incentive coupons to smooth drops in produce consumption at the end of the month. These findings also highlight trade-offs inherent in different delivery mechanisms for SNAP incentives.

The Supplemental Nutrition Assistance Program (SNAP) provides benefits to more than 41 million Americans, or 12% of the US population^(1)^. SNAP benefits can be spent on food, but SNAP participants mirror the rest of the US population in consuming low levels of healthy foods such as fruits and vegetables^(2)^. An increasingly common policy to address unhealthy diets among SNAP households is incentivising the purchase of healthier foods. Examples of policies are the federal Gus Schumacher Nutrition Incentive Program (GusNIP) and the Healthy Fluid Milk Incentive program. These incentives may either reduce the price for target food or give benefits for future spending when a household purchases target foods with SNAP. Providing incentives for healthier purchases can increase overall spending and intake on target foods^(3,4)^, though barriers to obtaining and using these incentives may be substantial^(5)^.

Beyond having low levels of healthy food consumption, the amount of healthy foods consumed by SNAP households varies substantially over time. SNAP benefits are issued to participating households once a month, after which households generally spend benefits quickly. As a result, overall food expenditures and consumption drops until the next monthly payment^(6–8)^. SNAP household diet quality similarly decreases over the month^(9)^, and SNAP households buy and consume fewer fruits and vegetables at the end of the SNAP month^(10)^. In addition to real changes in food consumption, households perceive greater food insecurity at the end of the SNAP month^(11)^. Household-level changes mask individual variation: food consumption drops at the end of the month less for children who have access to school meal programmes^(8)^. These changes in food consumption have real health impacts. Emergency room visits increase among elderly SNAP participants at the end of the SNAP month^(12)^, as do hospital admissions due to hypoglycaemia^(13)^. Intra-monthly declines in food consumption are likely caused by greater resource constraints at the end of the SNAP month, as crime is also sensitive to the timing of SNAP benefit payments^(14)^.

Food expenditure, consumption and related outcomes change at different rates over the SNAP month. Food expenditure drops immediately after benefit payment and stays at lower levels for the rest of the SNAP month^(8)^. Food consumption, however, decreases more steadily over the SNAP month, with the most noticeably lower overall food and fruit and vegetable intake occurring just before the next monthly payment^(9,10)^. Perhaps as a result of this concentration of lower consumption at the end of the SNAP month, many of the other changes also occur right at the end of the SNAP month as well^(12–14)^. Changes in behaviour, thus, often occur nonlinearly over the SNAP month.

While some is understood about the impact of SNAP incentive programmes overall, less is known about how SNAP incentives impact intra-month variation in SNAP household diets. Sruthi Valluri and colleagues^(15)^ examined a fruit and vegetable incentive programme for near-SNAP eligible households and found that households provided incentives had similar drops in fruit and vegetable spending as control households. In a survey of seniors at mobile markets in Rhode Island that provide SNAP incentives, respondents stated that their SNAP benefits lasted longer due to participation in the programme^(16)^. To our knowledge, no study has examined the potential for SNAP incentives to mitigate cyclical changes in diet quality among the broader SNAP population.

This article investigated SNAP household use of incentives over the month in the context of a GusNIP initiative in Alabama. Households that purchase fresh fruits and vegetables (FFV) using SNAP benefits at participating retailers receive a coupon that entitles them to obtain a matching amount of FFV. Households can use the coupons in any future transaction. We used data from the universe of SNAP transactions at three participating Alabama grocery stores to characterise SNAP and incentive spending on FFV over the month in relation to the Alabama SNAP issuance cycle.

## Methods

### Study setting and sample

In 2021, the Alabama GusNIP initiative started offering incentives in two independent grocery stores and seven farmer’s markets across Alabama. In 2022, the initiative expanded to three more independent grocery stores while one of the original grocery stores dropped out. Given the importance of grocery stores relative to farmers markets for SNAP households^(17)^, this article focuses on incentive use at the grocery stores. Whenever a household uses SNAP benefits to purchase FFV at any of the participating grocery stores, the store automatically issues an incentive, which is a paper coupon for the amount that the household had spent out of SNAP on FFV (a 1:1 match). Coupon amounts are capped at $10. Households can redeem the coupon at any time either as part of another transaction or alone. Coupons do not have unique identification numbers, so there is no way to track individual coupons across time.

We obtained transaction-level data from each store participating in the Alabama incentive programme for all SNAP or incentive transactions starting in 2021. SNAP transaction data provided summary information for each transaction in which SNAP benefits were used: the date, the amount of SNAP benefits used, the dollar amount of benefits spent on incentive-eligible FFV, the dollar amount of incentives issued and the dollar amount of incentives redeemed. For transactions that only redeemed an incentive, we obtained the date of the transaction and the dollar amount of incentives redeemed. Our initial sample consisted of 296 653 unique transactions. Since we were not able to track incentive use by individual households, we aggregated the data to the day level for each store.

Alabama issues SNAP benefits between the 4th and 23rd of each month. The specific day that a household receives benefits is determined by the last four digits of the household’s case number. Prior to April 2023, Alabama also issued pandemic emergency allotment amounts on the first of the month to all households. Prior literature has focused on the effect of once-per-month SNAP payments, and Emergency Allotments were a temporary policy, so we restricted our store-day sample to days between April 2023 and July 2023. One of the four participating grocery stores only provided partial data, so we excluded that store to ensure that the composition of stores does not change over time. To ensure that we look at the same number of days in each month, we also excluded observations corresponding to the 31 May and 31 July. Our final sample consists of 356 store-day observations from three grocery stores.

### Analysis

We used the transaction-level data to create four outcome variables describing daily SNAP FFV spending and incentive redemption for each store. We aggregated the daily transactions per store to obtain the total dollar amount of incentives redeemed in a store on a particular day, and the total dollar amount of incentive-eligible FFV purchased using SNAP on that day (including incentive redemptions). Our first outcome was the daily total amount of redemptions for that store (‘Redemptions’), and our second outcome was the daily total amount of SNAP FFV spending at that store (‘Total FFV Spending’).

We used these daily store totals to measure changes in the composition of SNAP and FFV spending over the month. Our third outcome was the daily percentage of total spending (SNAP plus incentives) that was spent on FFV at that store (‘% of total spending on FFV’). For our fourth outcome, we calculated the daily percentage of total FFV spending (SNAP plus incentives) that came from incentive redemptions at that store (‘% of FFV from redemptions’). To ease interpretation of the results, all percentages range from 0 to 100.

We estimated regressions of our outcome variables (redemptions, total FFV spending, % of total spending on FFV, and % of FFV from redemptions) on an indicator identifying whether a day is outside of the SNAP issuance window (either the 1st–3rd of a month or the 24th–30th). Regressions also controlled for month-fixed effects and store-fixed effects. Standard errors were clustered at the store level. Our main text shows results using traditional cluster-robust standard errors. Since our sample only has three stores, however, we show in the Appendix that our results did not appreciably change when we used the wild cluster bootstrap to calculate standard errors^(18)^.

A single indicator for being outside of the SNAP issuance window summarises spending outside of the window but masks how spending changes further from the end of the SNAP issuance window. In a secondary analysis, instead of the single indicator, we included indicators for whether the observation was 1–3 d after the end of the SNAP issuance window, 4–6 d after the end of the window and 7–10 d after the end of the window. All coefficients are interpreted relative to days within the SNAP issuance window. As above, we controlled for month and store-fixed effects, and standard errors were clustered at the store level. Wild cluster bootstrap results are shown in the Appendix.

## Results

### Supplemental Nutrition Assistance Program *and incentive redemptions over the* Supplemental Nutrition Assistance Program *month*

Table 1 summarises our outcome variables over the SNAP issuance window in the three stores between April and July 2023. For reference, we also include mean daily total store-level SNAP spending. Total SNAP spending drops by over half outside of the SNAP window relative to SNAP spending in the window, and total FFV spending drops by almost half. In contrast, redemptions only drop by $9·51 or 8%. Outside of the SNAP window, the percentage of spending going to FFV increases by 2·3 percentage points, and the percentage of FFV from redemptions increases by almost 10 percentage points. Outside of the SNAP window, almost a third of daily FFV spending is from incentive redemptions.

**Table 1:** Daily SNAP, FFV and incentive redemptions in stores participating in Alabama GusNIP, for days in the Alabama SNAP issuance window and days out of the SNAP issuance window, 2023

	In SNAP issuance window	Out of SNAP issuance window
Mean daily SNAP spending total, $	10 046·29	4738·91
	(3004·77)	(2074·47)
Mean total FFV spending, $	700·86	425·47
	(302·64)	(199·50)
Mean redemptions, $	119·07	109·56
	(58·66)	(47·76)
Mean % of total spending on FFV	6·90	9·20
	(1·71)	(2·52)
Mean % of FFV from redemptions	17·39	27·25
	(5·10)	(8·62)
No. observations (store days)	238	118

Notes: Table summarises daily transactions at three grocery stores April–July 2023. SNAP, Supplemental Nutrition Assistance Program; FFV, fresh fruits and vegetables.

Spending is daily store-level SNAP expenditures plus incentive redemption. Redemptions are daily store-level incentive redemptions in dollars. Total FFV spending is daily SNAP store-level spending in dollars on incentive-eligible fresh fruits and vegetables, including incentive redemptions. % of total spending on FFV is the percentage of total daily SNAP spending that goes to FFV. % of FFV from redemptions is the percentage of SNAP FFV spending that comes from redemptions.

**Fig. 1:**
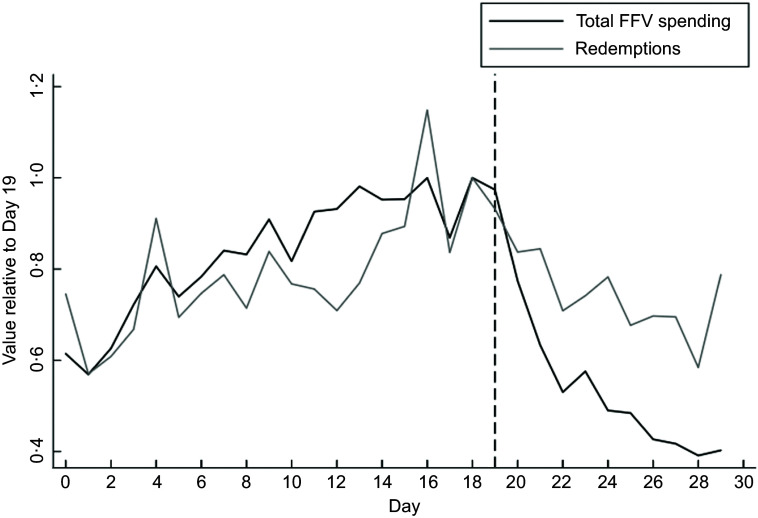
Mean daily total FFV spending and total redemptions for days relative to the start of the Alabama SNAP issuance window (Day = 0), 2023. FFV = incentive-eligible fresh fruits and vegetables. Total FFV spending is FFV spending using SNAP benefits plus incentive redemptions. Figure displays mean values of total FFV spending and total incentive redemption for each day of the month, normalised by the value in Day 19. Day 0 corresponds to the first day of the SNAP issuance window, and Day 19 corresponds to the end of the SNAP issuance window in Alabama. FFV, fresh fruits and vegetables; SNAP, Supplemental Nutrition Assistance Program.

Figure 1 shows the trends in redemptions and total FFV spending. In the figure, days are re-defined relative to the start of the Alabama SNAP issuance period: Day 0 corresponds to the 4th of the month and Day 29 corresponds to the 3rd of the next calendar month. Day 19 is thus the 23rd of the month, i.e. the end of the SNAP issuance window. To facilitate comparing trends over the SNAP issuance window, we normalised the average outcome for each day by the average outcome for Day 19. While FFV spending drops sharply in stores just after the end of the Alabama SNAP issuance window, incentive redemption does not.

**Fig. 2:**
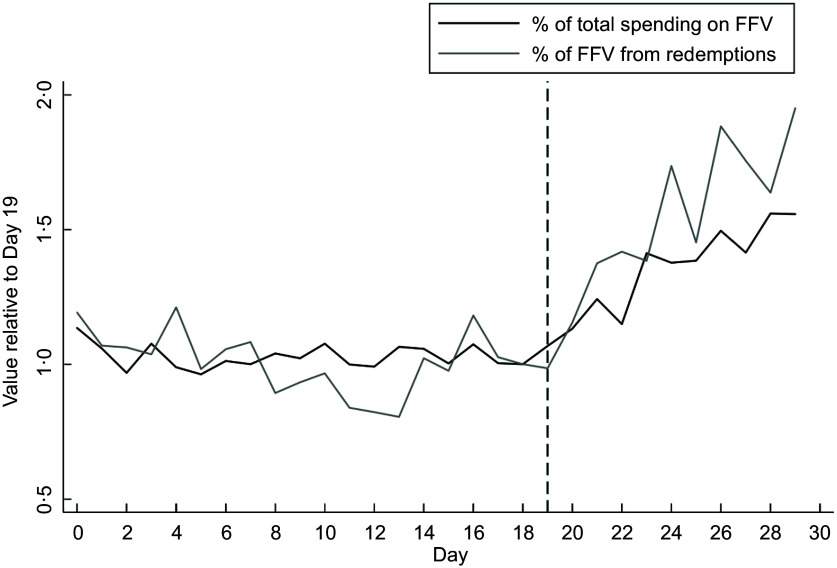
Mean daily % of SNAP spending going to incentive-eligible fresh fruits and vegetables (FFV) and mean daily % of FFV spending coming from redemptions, for days relative to the start of the Alabama SNAP issuance window (Day = 0), 2023. FFV = incentive-eligible fresh fruits and vegetables. Figure displays mean values of the % of SNAP spending to FFV and % of FFV spending from incentive redemptions for each day of the month, normalised by the value in Day 19. Day 0 corresponds to the first day of the SNAP issuance window, and Day 19 corresponds to the end of the SNAP issuance window in Alabama. SNAP, Supplemental Nutrition Assistance Program; FFV, fresh fruits and vegetables.

In Fig. 2, we show the implications of incentive redemptions for trends in the % of total spending on FFV and % of FFV from redemptions over the SNAP issuance window. As in Fig. 1, to highlight the trends over time, we normalised values by the value at the end of the SNAP issuance window (Day 19). As soon as the SNAP issuance window ends, the percentage of spending on FFV starts to rise. Similarly, the percentage of FFV spending that comes from incentives increases steadily as soon as the SNAP issuance window ends.

Table 2 displays the results of the regressions. Full regression output is given in the online Appendix. The top panel of Table 2 shows the coefficient on the indicator for whether the store-day is outside of the SNAP window, and the bottom panel shows coefficients from the secondary analysis measuring the outcome over time since the end of the SNAP window. As suggested in Figs. 1 and 2, there is no statistically significant drop in daily incentive redemptions after the end of the SNAP issuance window. Daily total store SNAP FFV spending is almost $300 lower outside of the SNAP window, representing a 39% drop relative to the mean FFV spending in the SNAP window. Outside the SNAP window, stores see a 2 percentage point increase in the percent of SNAP spending going to FFV relative to within the SNAP window. The percentage of FFV spending coming from incentives increases by almost 10 percentage points outside the SNAP window relative to within the SNAP window.

**Table 2:** Change in redemptions, total FFV spending, percentage of total spending on FFV and percentage of FFV from redemptions outside of the SNAP issuance window relative to inside of the SNAP issuance window, 2023

	Outcome			
Variable	Redemptions, $	Total FFV spending, $	% of total spending on FFV	% of FFV from redemptions
*Outside of SNAP issuance window (v. within window)*				
Out of window	–8·903	–272·2*	2·321***	9·864***
	(9·703)	(73·15)	(0·186)	(0·531)
*Number of days after end of SNAP issuance window (v. inside of SNAP issuance window)*				
1–3 d	1·085	–155·2*	1·004**	5·289**
	(4·538)	(46·34)	(0·145)	(0·642)
4–6 d	–8·728	–266·7*	2·448***	9·008**
	(12·26)	(72·64)	(0·225)	(1·242)
7–10 d	–16·31	–361·3*	3·187**	13·82***
	(12·33)	(92·33)	(0·384)	(0·611)
N	356	356	356	356

Source: Authors’ analysis of daily grocery store transaction data.

FFV, fresh fruits and vegetables; SNAP, Supplemental Nutrition Assistance Program.

Spending is daily store-level SNAP expenditures plus incentive redemption. Redemptions are incentive redemptions in dollars. Total FFV spending is daily SNAP store-level spending in dollars on incentive-eligible fresh fruits and vegetables, including incentive redemptions. % of total spending on FFV is the percentage of total daily SNAP spending that goes to FFV. % of FFV from redemptions is the percentage of SNAP FFV spending that comes from redemptions.

* *P* < 0.10.

** *P* < 0.05.

*** *P* < 0.01.

The drop in FFV and relative importance of incentives increases as the time since the end of the SNAP issuance window increases. Relative to inside the SNAP issuance window, daily total FFV spending is around $360 lower 7–10 d after the end of the window, representing a drop of 51% in total FFV spending. Relative to inside the SNAP issuance window, the percent of total spending going to FFV increases by 3·2 percentage points, and the percent of FFV spending from incentives increases by 13·8 percentage points 7–10 d after the end of the issuance window. Far from the SNAP issuance window, thus, the share of spending going to FFV increases by 46% and the share of FFV spending from incentives increases by almost 80% relative to means within the SNAP issuance window.

Our main analyses use every day of the month. Some regular payments (e.g. paychecks, Social Security benefits) get paid on the first of the month, and daily grocery shopping trips might be influenced by these other payments. In Appendix Table 4, we show our regression results after dropping transactions on the first of the month. Our results do not appreciably change, suggesting that our findings are driven more by the SNAP issuance window than the timing of other payments.

## Discussion

This article used daily store-level transaction data to measure how SNAP households use incentive coupons throughout the month. We examine intra-monthly SNAP spending in the context of a SNAP nutrition incentive programme that provides SNAP households with coupons to use on FFV. We found that, in contrast to SNAP spending on FFV, incentive redemptions are spent evenly throughout the month. Additionally, a greater share of daily SNAP FFV spending at stores comes from incentives after SNAP stops being issued each month. Perhaps partly due to incentives, the percentage of total SNAP spending that goes to FFV increases after SNAP is no longer issued each month. Our results thus demonstrate that households are more likely to use incentive coupons when they have fewer other resources to purchase foods, such as at the end of the month. When provided as coupons for future use, incentives therefore have the potential to mitigate the drop in diet quality experienced by SNAP households in between SNAP benefit payments.

Since GusNIP provides money to local initiatives, the contours of SNAP incentive programmes differ greatly across initiatives. Some GusNIP initiatives provide incentives in only farmers markets. Other initiatives target grocery stores, while others target both farmers markets and grocery stores. In many GusNIP initiatives, SNAP participants earn points or coupons for future use when they use SNAP to purchase eligible food items^(4)^. Alternatively, incentives could be provided as an immediate reduction in the price of the product^(19)^. While prior studies have examined the impact of individual incentive programmes on outcomes such as spending or diet, less is known about the trade-offs inherent in the design of the programmes. One study in California looked at the impacts on diet of a SNAP-like voucher programme that either restricted eligible items or provided incentive vouchers weekly instead of monthly for 6 months^(20)^. The study found that neither restricting eligible items nor providing vouchers more frequently influenced the impact of the programme on diet at the end of the 6 months. We build on this study by examining how incentive programmes that provide coupons for future use can smooth intra-monthly spending variation.

We note that our study has limitations. We do not have access to data on control stores that do not offer incentives and are thus unable to measure the extent to which incentives cause any changes in intra-month spending. Incentive-related changes to spending could come from the intensive margin on any particular trip – increased FFV spending when the household already was purchasing FFV – or from the extensive margin – the household buying FFV on a trip when they would not have otherwise purchased FFV. Our results could be driven by incentive-related changes to daily spending on the intensive or extensive margins. We therefore are not able to measure counterfactual spending in the absence of incentives.

In addition, our transaction-level data provide a lot of information but exclude information that would provide a more detailed picture of how households use coupons. Importantly, we do not consistently have household identifiers for both SNAP transactions and coupon redemptions. We therefore cannot measure how long households hold on to coupons and cannot match a household’s coupon redemption to that household’s SNAP issuance. This necessitates a store-level analysis as we do here, instead of a household-level analysis. In addition, our data only include SNAP spending on FFV. We are thus unable to measure how coupon use is related to non-SNAP FFV spending. Transaction data from each store also only provides information on household spending and not the timing or composition of consumption. FFV have limited shelf-life, so many of the items obtained using the incentives could not be stored over a long period of time. However, if the items obtained using incentives expire or are wasted, consumption may be less than spending.

This study is necessarily focussed on three independent grocers in Alabama. These grocers are smaller than other (especially chain) grocery stores, and spending at these stores may not represent overall SNAP spending. In particular, we cannot rule out that households spend differently at other stores. Since incentives were only redeemable at the participating grocery stores, this limitation does not apply to our characterisation of incentive redemptions. We are, however, unable to observe the true total SNAP benefits spent by each household. Our results also may not generalise to larger grocery stores or to stores outside of Alabama. Furthermore, our results may not generalise to SNAP populations outside of Alabama. SNAP households in Alabama are poorer, have more children and are more likely to consist of single parents than SNAP households nationwide (Appendix Table 5). In addition, during the period of our data, Alabama issued pandemic Electronic Benefit Transfer (EBT). Households with children in day care were eligible for benefits throughout the year, and households with school-aged children received a lump sum of $120 per child as summer pandemic EBT. These additional payments may limit the generalisability of our study results to other time periods.

Despite these limitations, our results have important implications for current SNAP incentive policy. Recently, the USDA has shown interest in providing incentives in the form of price discounts at the point of sale instead of coupons. Proposals for the Healthy Fluid Milk Incentive programme, for example, now only allow for projects that provide immediate discounts or deposit earned incentives automatically onto SNAP cards^(21)^. This incentive mechanism can be expected to increase the use of incentives, but incentives provided as immediate discounts may not be as successful at smoothing consumption of FFV across the month. Our results therefore highlight the trade-offs inherent in the choice of the incentive mechanism.

Our study also points to important areas for future work. Matching individual redemptions to households would allow for a more detailed analysis of household incentive use. In particular, it would be important to understand how long a household takes to use a coupon (if they ever use it) and what factors influence waiting to use coupons. This study was restricted to three grocers in Alabama that participated in the incentive programme, and thus we were unable to measure the extent to which households substituted between stores. Future work also should use a wider range of months to investigate whether incentive use changes over the year. In addition, as programmes transition to automatic discounts at the point of sale, future research will be needed to understand the benefits and drawbacks for SNAP households of different incentive delivery mechanisms.

SNAP households use healthy eating incentives differently across the month, and use of incentives may smooth consumption of FFV over time. Our findings suggest trade-offs between different incentive delivery mechanisms, as the ability to use incentives differently across time depends on how incentives are provided.

## Supporting information

Cuffey et al. supplementary material Cuffey et al. supplementary material

## Supplementary material

For supplementary material accompanying this paper visit https://doi.org/10.1017/S1368980024001770
