# Supplementary material for: Supplemental Nutrition Assistance Program healthy eating incentives and fruit and vegetable spending across the month
Source: Public Health Nutr. 2024 Sep 26;27(1):e178. doi: 10.1017/S1368980024001770 (PMC11504429; doi:10.1017/S1368980024001770)
Supplement: Cuffey et al. supplementary material [file S1368980024001770sup001.docx]

**Online Appendix**

Appendix Table 1: Spending on FFV and redemptions outside of the SNAP issuance window relative to inside of the SNAP issuance window (detailed regression output)

|  | Outcome | | | |
| --- | --- | --- | --- | --- |
| Variable | Redemptions, $ | Total FFV spending, $ | % of total spending on FFV | % of FFV from redemptions |
|  |  |  |  |  |
| Out of window | -8.903 | -272.2^*^ | 2.321^***^ | 9.864^***^ |
|  | (9.703) | (73.15) | (0.186) | (0.531) |
|  |  |  |  |  |
| Month (ref: April) |  |  |  |  |
| May | -10.48 | -23.95 | 0.0122 | -0.866 |
|  | (6.191) | (23.85) | (0.475) | (0.648) |
| June | 4.914 | 15.22 | 0.333 | 1.256^**^ |
|  | (7.957) | (42.80) | (0.594) | (0.275) |
| July | -3.359 | -39.57^*^ | -0.560^*^ | 1.665 |
|  | (6.885) | (9.627) | (0.154) | (1.557) |
|  |  |  |  |  |
| Adj R2 | 0.346 | 0.597 | 0.602 | 0.359 |
|  |  |  |  |  |
| N | 356 | 356 | 356 | 356 |

Source: Authors’ analysis of daily grocery store transaction data. Notes: FFV=fresh fruits and vegetables. Spending is daily store-level SNAP expenditures plus incentive redemption. Redemptions is incentive redemptions in dollars. Total FFV spending is daily SNAP store-level spending in dollars on incentive-eligible fresh fruits and vegetables, including incentive redemptions. % of total spending on FFV is the percentage of total daily SNAP spending that goes to FFV. % of FFV from redemptions is the percentage of SNAP FFV spending that comes from redemptions. * *p* < 0.10, ** *p* < 0.05, *** *p* < 0.01.

Appendix Table 2: Spending on FFV and incentive use over days outside of the SNAP issuance window relative to inside of the SNAP issuance window (detailed regression output)

|  | Outcome | | | |
| --- | --- | --- | --- | --- |
| Variable | Redemptions, $ | Total FFV spending, $ | % of total spending on FFV | % of FFV from redemptions |
| *Number of days after end of SNAP issuance window (vs. inside of SNAP issuance window)* | | | | |
| 1-3 days | 1.085 | -155.2^*^ | 1.004^**^ | 5.289^**^ |
|  | (4.538) | (46.34) | (0.145) | (0.642) |
| 4-6 days | -8.728 | -266.7^*^ | 2.448^***^ | 9.008^**^ |
|  | (12.26) | (72.64) | (0.225) | (1.242) |
| 7-10 days | -16.31 | -361.3^*^ | 3.187^**^ | 13.82^***^ |
|  | (12.33) | (92.33) | (0.384) | (0.611) |
|  |  |  |  |  |
| Month (ref: April) |  |  |  |  |
| May | -10.48 | -23.95 | 0.0122 | -0.866 |
|  | (6.208) | (23.90) | (0.476) | (0.650) |
| June | 4.914 | 15.23 | 0.332 | 1.256^**^ |
|  | (7.979) | (42.91) | (0.596) | (0.275) |
| July | -3.243 | -38.17^**^ | -0.574^*^ | 1.603 |
|  | (6.785) | (8.112) | (0.144) | (1.593) |
|  |  |  |  |  |
| Adj R2 | 0.348 | 0.622 | 0.653 | 0.423 |
|  |  |  |  |  |
| N | 356 | 356 | 356 | 356 |

Source: Authors’ analysis of daily grocery store transaction data. Notes: FFV=fresh fruits and vegetables. Spending is daily store-level SNAP expenditures plus incentive redemption. Redemptions is incentive redemptions in dollars. Total FFV spending is daily SNAP store-level spending in dollars on incentive-eligible fresh fruits and vegetables, including incentive redemptions. % of total spending on FFV is the percentage of total daily SNAP spending that goes to FFV. % of FFV from redemptions is the percentage of SNAP FFV spending that comes from redemptions. * *p* < 0.10, ** *p* < 0.05, *** *p* < 0.01.

Appendix Table 3: Spending on FFV and incentive use outside of the SNAP issuance window relative to inside of the SNAP issuance window (wild cluster bootstrap)

|  | Outcome | | | |
| --- | --- | --- | --- | --- |
| Variable | Redemptions, $ | Total FFV spending, $ | % of total spending on FFV | % of FFV from redemptions |
|  |  |  |  |  |
| *Outside of SNAP issuance window (vs. within window)* | | | | |
| Out of window | -8.903 | -272.2^*^ | 2.321^**^ | 9.864^***^ |
|  | [0.3615] | [0.0832] | [0.0328] | [0.0372] |
|  |  |  |  |  |
| *Number of days after end of SNAP issuance window (vs. inside of SNAP issuance window)* | | | | |
| 1-3 days | 1.085 | -155.2^*^ | 1.004 | 5.289^*^ |
|  | [0.8100] | [0.0740] | [0.1024] | [0.0568] |
| 4-6 days | -8.728 | -266.7^*^ | 2.448^*^ | 9.008^*^ |
|  | [0.4309] | [0.0942] | [0.0530] | [0.0604] |
| 7-10 days | -16.31 | -361.3^*^ | 3.187^*^ | 13.82^***^ |
|  | [0.3539] | [0.0956] | [0.0608] | [0.0088] |
| N | 356 | 356 | 356 | 356 |

Source: Authors’ analysis of grocery store transaction data. Notes: Wild cluster-robust p-values in brackets below coefficients. P-values are from wild cluster bootstrap standard errors, with clustering by store. FFV=fresh fruits and vegetables. Spending is daily store-level SNAP expenditures plus incentive redemption. Redemptions is incentive redemptions in dollars. Total FFV spending is daily SNAP store-level spending in dollars on incentive-eligible fresh fruits and vegetables, including incentive redemptions. % of total spending on FFV is the percentage of total daily SNAP spending that goes to FFV. % of FFV from redemptions is the percentage of SNAP FFV spending that comes from redemptions. * *p* < 0.10, ** *p* < 0.05, *** *p* < 0.01.

Appendix Table 4: Change in redemptions, total FFV spending, percentage of total spending on FFV, and percentage of FFV from redemptions, outside of the SNAP issuance window relative to inside of the SNAP issuance window, 2023 – Excluding the first of the month

|  | Outcome | | | |
| --- | --- | --- | --- | --- |
| Variable | Redemptions, $ | Total FFV spending, $ | % of total spending on FFV | % of FFV from redemptions |
|  |  |  |  |  |
| *Outside of SNAP issuance window (vs. within window)* | | | | |
| Out of window | -8.140 | -262.7^*^ | 2.294^***^ | 9.512^***^ |
|  | (9.783) | (71.37) | (0.185) | (0.708) |
|  |  |  |  |  |
| *Number of days after end of SNAP issuance window (vs. inside of SNAP issuance window)* | | | | |
| 1-3 days | 1.073 | -155.1^*^ | 1.006^**^ | 5.283^**^ |
|  | (4.533) | (46.37) | (0.144) | (0.643) |
| 4-6 days | -8.740 | -266.7^*^ | 2.449^***^ | 9.002^**^ |
|  | (12.27) | (72.67) | (0.226) | (1.246) |
| 7-10 days | -16.51 | -363.4^*^ | 3.394^**^ | 14.12^***^ |
|  | (13.37) | (93.13) | (0.445) | (1.139) |
| N | 344 | 344 | 344 | 344 |

Source: Authors’ analysis of daily grocery store transaction data. Notes: FFV=fresh fruits and vegetables. Spending is daily store-level SNAP expenditures plus DUFB redemption. Redemptions is incentive redemptions in dollars. Total FFV spending is daily SNAP store-level spending in dollars on incentive-eligible fresh fruits and vegetables, including incentive redemptions. % of total spending on FFV is the percentage of total daily SNAP spending that goes to FFV. % of FFV from redemptions is the percentage of SNAP FFV spending that comes from redemptions. * *p* < 0.10, ** *p* < 0.05, *** *p* < 0.01.

Appendix Table 5: Mean characteristics of SNAP participants, 2020 pre-pandemic

|  | Alabama | US |
| --- | --- | --- |
| Income as % of poverty | 58.6 | 64.5 |
|  |  |  |
| Monthly SNAP benefit ($) | 241 | 230 |
|  |  |  |
| Household size (# individuals) | 2.1 | 1.9 |
|  |  |  |
| % of households with children | 45.3 | 38.1 |
|  |  |  |
| % of households with elderly | 23.4 | 28.6 |
|  |  |  |
| % of households single adults with children | 35.6 | 23.9 |
|  |  |  |
| % of households with able-bodied adults and no children | 10.3 | 13.4 |

Source: “Characteristics of Supplemental Nutrition Assistance Program Households: Fiscal Year 2020” (USDA Office of Policy Support Report No. SNAP-21-CHAR).
